# Supplementary material for: Germline pathogenic variants of 11 breast cancer genes in 7,051 Japanese patients and 11,241 controls
Source: Nat Commun. 2018 Oct 4;9:4083. doi: 10.1038/s41467-018-06581-8 (PMC6172276; doi:10.1038/s41467-018-06581-8)
Supplement: Supplementary file 1 — Supplementary Information [file 41467_2018_6581_MOESM1_ESM.pdf]

Supplementary Information

Germline pathogenic variants of 11 breast cancer genes in 7,051 Japanese patients and 11,241 controls

Authors: Yukihide Momozawa[1,*], Yusuke Iwasaki[1], Michael T. Parsons[2], Yoichiro Kamatani[3], Atsushi Takahashi[3,4], Chieko Tamura[5], Toyomasa Katagiri[6], Teruhiko Yoshida[7], Seigo Nakamura[8], Kokichi Sugano[7,9], Yoshio Miki[10], Makoto Hirata[11], Koichi Matsuda[12], Amanda B. Spurdle[2], Michiaki Kubo[1,*]

Contents

Supplementary Note 1-4	2
Supplementary Tables 1-16	9
Supplementary Fig. 1-6	25
Supplementary References	31

Supplementary Note

Supplementary Note 1. Determination of clinical significance of each variant

Sequencing of the 11 established hereditary breast cancer genes identified 1,781 germline variants among 7,051 breast cancer cases and 11,241 controls. According to the genomic position, we categorized the variants into 210 disruptive, 1,084 nonsynonymous, and 487 synonymous variants (Supplementary Table 8). Minor allele frequencies (MAF) of these variants in controls were common (MAF ≥ 5%) for 30 variants, low (5% > MAF ≥ 1%) for 27 variants, and rare (MAF < 1%) for 1,724 variants. More than half of the variants (all rare) were not registered at dbSNP147[1]. When we examined the density of variants in each gene, the number of variants was strongly correlated with the gene length (Pearson correlation coefficient, r = 0.953, p = 5.70 x 10^{-6}, Supplementary Fig. 6).

In ClinGen[2], expert panels of *BRCA1/2*, *CDH1*, *PTEN*, and *TP53* work to establish robust variant curation rules and processes. However, there is no expert panel for the other genes tested in this study. Therefore, to maintain consistency of variant annotation across the 11 hereditary breast cancer genes, we decided to use the American College of Medical Genetics and Genomics and the Association for Molecular Pathology (ACMG/AMP) guidelines[3] to assess all 11 genes analyzed in this study. The ACMG/AMP guidelines provided a standardized framework for evaluation of the clinical significance of variants, but practical methodologies for variant classification were not included[4]. We determined practical methodologies for each criterion (Supplementary Table 9, 10) partially based on the knowledge of variant classification guidelines

developed by the Evidence-based Network for the Interpretation of Germline Mutant Alleles (ENIGMA) consortium[5] for *BRCA1/2*.

We first applied two sets of criteria for each variant, one for classification of pathogenic variants (PVS1, PS1-4, PM1-6, and PP1-5) and the other for classification of benign variants (BA1, BS1-4, and BP1-7), with some modification because the Biobank Japan did not collect parent samples or information about segregation of variants in affected family members. We checked every variant using case-control association results in this study, known clinical significance information from ClinVar[6], population data from the 1000 genomes project[7], ExAC[8], and Tohoku Medical Megabank Organization (ToMMo)[9], computational predictions using in silico programs such as FATHMM (ver. 3.0a)[10], KGGSeq (ver. 1.0+)[11], and SPIDEX (ver. 1.0)[12], and functional data summarized by the Human Gene Mutation Database Pro (ver. 2016.1)[13]. The effect of each variant on protein sequence was predicted using SnpEff (ver. 4.2)[14] with all transcripts registered in CCDS release 15 (Supplementary Table 7)[15]. Since some genes have multiple transcripts, the highest effect predicted by SnpEff was used[16]. When a variant had two or more protein positions caused by multiple transcripts, protein position was reported based on the longest transcript throughout the manuscript. Annotation based on all transcripts is shown in Supplementary Data 1 for women and Supplementary Data 2 for men.

For each variant, the pathogenic and benign sets of criteria were combined according to the scoring rules of ACMG/AMP guidelines, and the variant classified into one of five categories: “pathogenic”, “likely pathogenic”, “likely benign”, “benign”, and “uncertain significance”. Uncertain significance was

assigned when both pathogenic and benign evidence existed (i.e. there was conflicting information for a given variant) or when not enough evidence existed to reach another category. In women, we initially classified 224 variants as pathogenic, 227 as benign and 1,330 as variants of uncertain significance (VUS). We also classified 66 as pathogenic, 176 as benign, and 1,028 as VUS in men. Next, we compared the clinical significance of each variant annotated in this study with the annotations recorded in the ClinVar database[6] (Supplementary Table 11). There was no apparent controversy between classification assigned by this study and that recorded in ClinVar eg pathogenic in this study versus benign in ClinVar. Among 1,781 variants found in unselected Japanese female breast cancer cases and controls, 768 variants were registered in ClinVar: 113 as pathogenic, 246 as benign, and 409 as VUS.

Among the 113 pathogenic variants registered in ClinVar, using data from our study alone, 93 (82.3%) were classified as pathogenic and the remaining 20 variants were classified as VUS because of insufficient evidence (Supplementary Table 12). We checked review status and evidence of these 20 variants that showed “conflicting” classification, and our investigations supported the assignment of these 20 variants as pathogenic based on information available outside of our study. Namely: 6 variants in *BRCA1* and *BRCA2* were reviewed by expert panel; 10 variants were submitted by multiplex submitters without conflicting interpretations; supporting evidence of pathogenicity was reported in ClinVar for two variants (p.Arg267Trp in *TP53* and p.Asn2387_Phe2388del in *NF1*); additional evidence in support of pathogenicity provided by the submitter, Ambry Genetics, for two other variants (Tina Pesaran, personal communication),

namely *PTEN* p.Tyr68Cys was classified pathogenic because it met ACMG/AMP criteria PS3, PM1, PM2, PM5, PP3, and PP4, while *BRCA2* p.Thr2722Lys was likely pathogenic because it met ACMG/AMP PM1, PM2, PM5, and BP4 criteria.

Among 246 variants registered as benign in ClinVar, 117 (47.7%) variants were also benign in our study and 129 (52.4%) variants were VUS. We considered all 246 variants as benign. Among 409 variants registered as VUS in ClinVar, information available from our study allowed us to classify 18 variants as pathogenic and 79 variants as benign. An additional 1,013 variants were not registered in ClinVar. Among them, 131 variants were classified as pathogenic and 110 variants as benign based on the clinical significance annotated in this study. Finally, the number of pathogenic, benign, and VUS was 244, 356, and 1,181, respectively (Supplementary Data 1).

We applied the same procedure to 1,270 variants found in male breast cancer cases and controls (Supplementary Table 13). We finally classified these variants as pathogenic for 75 variants, benign for 296, and VUS for 899, respectively (Supplementary Data 2).

Supplementary Note 2. Selection of patients according to NCCN guidelines

We selected patients by the National Comprehensive Cancer Network (NCCN) guidelines for genetic/familial high-risk assessment of breast and/or ovarian cancer (ver. 2.2016) [17] for the comparison of proportion of patients with a pathogenic variant with another study[18] because they selected patients based on the NCCN guidelines. Since Biobank Japan did not collect clinical information of breast cancer as hereditary disease, we did not have some information for family

members (a known mutation of hereditary breast cancer genes within the family, and age at diagnosis of breast cancer and histology of ovarian cancer in close relatives). Thus, we slightly modified the criteria as follows. (1) Age at breast cancer diagnosis ≤ 50 years old, (2) triple negative breast cancer diagnosed at ≤ 60 years old, (3) bilateral breast cancer, (4) comorbidity of pancreatic cancer at any age, (5) ≥ 1 family member with breast cancer at any age (not ≤ 50 years), (6) ≥ 1 family member with ovarian cancer (not invasive ovarian cancer) at any age, and (7) ≥ 2 family members with breast and/or pancreatic cancer at any age. Patients who met at least one of the criteria were treated as high-risk for further genetic risk evaluation. Patients who did not meet any of the criteria were considered as low-risk. If patients were not classified either high-risk or low-risk due to insufficient clinical information, they were considered as undetermined-risk. As results, 3,136 patients were high-risk, 1,164 were low-risk and 2,751 were undetermined-risk (Supplementary Table 14). The 3,136 high-risk patients were used for the comparison of proportion of patients with a pathogenic variant.

Supplementary Note 3. Influence of combining female and male controls

In this study, we analyzed women and men separately, as genetic risk for hereditary breast cancer differs between men and women[19]. However, there is a possibility to assign more variants as pathogenic by use of both female and male controls because the number of controls increases twofold from 11,241 to 23,731. To test this possibility, we combined both controls and determined clinical significance of all variants again. First, we focused the 1,781 variants found in women to check how the increased number of controls improved the

determination of clinical significance. As in the Supplementary Table 15, we observed that only one variant (p.Leu3048Phe in *ATM*) changed from “uncertain significance” to “pathogenic” because this variant came to meet PS4 of the ACMG/AMP guidelines. As a result, the combining female and male controls did not change the pathogenicity of many variants.

Then, we performed gene-based analysis with 245 pathogenic variants found in women and 39 additional pathogenic variants found in only male controls in 7,051 cases and 23,731 female and male controls (Supplementary Table 16). As a whole, results were very similar to Table 2 analyzed in 7,051 cases and 11,241 female controls only. However, when we checked each gene separately, we observed that odds ratio of *BRCA1* largely decreased from 33.0 to 20.5 because the frequency of controls with pathogenic variants increased from 0.04% to 0.07% by adding male controls. Among controls, men had more pathogenic variants in *BRCA1* (0.1%) than women (0.04%). This result is consistent with the recent publication about male breast cancer[19] which showed *BRCA1* was a low-risk gene (OR = 1.8). Therefore, female disease risk of *BRCA1* would be underestimated.

These results suggest that combining male and female controls would introduce bias of disease risk estimation when disease risk of a gene is different between both sexes.

Supplementary Note 4. Comparison of variant frequency in controls.

This study analyzed 11,241 female controls, but other studies have used data from the Exome Aggregation Consortium (ExAC)[8] as a control for the

estimation of disease risk[20]. We investigated the difference in allele frequency between Japanese women in this study and East Asian (EAS) and non-Finnish European (NFE) populations from ExAC without the Cancer Genome Atlas samples. We focused on rare variants with MAF <0.01 because all pathogenic variants were rare. In this study, we identified 1,724 rare variants, of which 1,011 (58.6%) were polymorphic in the controls and the remaining 713 variants were identified only in cases. However, only 87 (5.0%) and 31 (1.8%) were found in the EAS and NFE populations of ExAC, respectively. The frequency of relevant controls is indispensable for assigning clinical significance at PS4 of the ACMG/AMP guidelines and for estimating disease risk. However, because most rare variants were not found in ExAC, population-matched controls are necessary for appropriate assignment of clinical significance and better estimation of disease risk.

Supplementary Table 1: Characteristics of study population in men

Variable		Breast cancer patients		Controls[*1]	
No. of subjects		53		12,520	
Age at entry (Mean ± SD)		65.3±10.8		70.4±7.0	
Age at diagnosis (Mean ± SD)		61.5±11.6		-	
Family history of breast cancer	Yes	8	15.1%	0	0.0%
	No	45		12,520	
Family history of ovarian cancer	Yes	2	3.8%	0	0.0%
	No	51		12,520	
Family history of pancreas cancer	Yes	2	3.8%	0	0.0%
	No	51		12,520	
Family history of prostate cancer	Yes	2	3.8%	0	0.0%
	No	51		12,520	
Family history of thyroid cancer	Yes	1	1.9%	0	0.0%
	No	52		12,520	

Family history of cancer refers to reported cancer in first and/or second-degree relative.
[*1]Controls without past history nor family history of cancers were selected for this study.

Supplementary Table 2: Number of variants categorized as pathogenic, benign and uncertain significance in 11 genes for hereditary breast cancer.

Gene	Pathogenic		Benign		Uncertain		Sum
BRCA2	85	20.0%	78	18.3%	263	61.7%	426
BRCA1	55	24.3%	44	19.5%	127	56.2%	226
ATM	27	8.7%	53	17.0%	231	74.3%	311
PALB2	21	13.6%	30	19.5%	103	66.9%	154
CHEK2	17	21.0%	13	16.0%	51	63.0%	81
TP53	13	22.4%	12	20.7%	33	56.9%	58
PTEN	12	33.3%	4	11.1%	20	55.6%	36
NF1	8	3.6%	51	22.9%	164	73.5%	223
NBN	3	3.9%	20	26.3%	53	69.7%	76
CDH1	2	1.7%	31	27.0%	82	71.3%	115
STK11	1	1.3%	20	26.7%	54	72.0%	75
Sum	244	13.7%	356	20.0%	1,181	66.3%	1,781

Supplementary Table 3: Clinical information for double carrier women

Individual number	Pathogenic variant 1	Pathogenic variant 2	Clinical characteristics / note
(1)	p.Ile917fs in *BRCA1*	p.Lys918fs in *BRCA1*	Since both variants were located in the same haplotype, she was considered a single carrier of a pathogenic variant in *BRCA1* .
2	p.Gly2529fs in *BRCA2*	p.Ala523Thr in *CHEK2*	Age at diagnosis was 44. There was no history of other cancer but her mother also had breast cancer. Cancer was found in one side. Histological classifications were missed. TNM clinical classification was missed. Both estrogen and progesterone receptor status were positive.
3	p.Ala938fs in *BRCA2*	c.72+1G>A in *ATM*	Age at diagnosis was 45. There was no history of other cancer but her family member had lung cancer. Cancer was found in one side. Histological classification was the solid-tubular subtype of invasive ductal breast carcinoma. TNM clinical classifications were T1, N1, and M0. Both estrogen and progesterone receptor status were positive and HER2 was negative. She died at 50 years old.
4	p.Arg3128* in *BRCA2*	c.573+1delG in *CHEK2*	Age at diagnosis was 56. There was no history of other cancer but her family members had gastric cancer and testicular tumor. Cancer was found in one side. Histological classification was the solid-tubular subtype of invasive ductal breast carcinoma. TNM clinical classification were T2, N1, and M0. Hormone receptor status for estrogen, progesterone, and HER2 were positive, negative, and equivocal, repectively.

Supplementary Table 4: Comparison of clinical charactristics of breast cancer patients with and without pathogenic variants

Variables		Patients with pathogenic variants		Patients without pathogenic variants		P value	OR (95% CI)
No. of subjects		404		6,647			
Age at entry	years old	55.1 ± 12.7 (404)		59.4 ± 11.9 (6,645)		1.00E-10	
Age at diagnosis	years old	51.4 ± 12.8 (393)		56.1 ± 11.9 (6,240)		3.44E-12	
History of ovarian cancer	Yes	7	1.7%	40	0.6%	0.017	2.9 (1.1-6.6)
	No	397		6,607			
Location of cancer*	Both sides	20	7.1%	104	2.4%	6.11.E-05	3.1 (1.8-5.1)
	One side	262		4,157			
TNM clinical classification: N*	0	142	64.5%	2,566	75.8%	3.48E-04	
	1	62	28.2%	682	20.2%		
	2	14	6.4%	80	2.4%		
	3	2	0.9%	55	1.6%		
TNM clinical classification: M*	0	182		2,999			
	1	11	5.7%	72	2.3%	8.79.E-03	2.5 (1.2-4.9)
Estrogen-receptor status*	Positive	178	66.9%	3,360	73.3%	0.028	0.7 (0.6-1.0)
	Negative	88		1,225			
Progesterone-receptor status*	Positive	125	47.7%	2,788	61.8%	8.45.E-06	0.6 (0.4-0.7)
	Negative	137		1,725			
Triple negative breast cancer*	Yes	36	22.0%	297	10.1%	2.16.E-05	2.5 (1.6-3.7)
	No	128		2,638			
Family history of breast cancer	Yes	94	23.3%	740	11.1%	3.14.E-11	2.4 (1.9-3.1)
	No	310		5,907			
Family history of ovarian cancer	Yes	19	4.7%	64	1.0%	1.42.E-07	5.1 (2.8-8.7)
	No	385		6,583			
Family history of pancreas cancer	Yes	24	5.9%	220	3.3%	0.011	1.8 (1.1-2.9)
	No	380		6,427			
Family history of gastric cancer	Yes	101	25.0%	1,353	20.4%	0.027	1.3 (1.0-1.7)
	No	303		5,294			
Family history of liver cancer	Yes	38	9.4%	418	6.3%	0.017	1.5 (1.1-2.2)
	No	366		6,229			
Family history of bone tumor	Yes	4	1.0%	13	0.2%	0.014	5.1 (1.2-16.6)
	No	400		6,634			
Family history of bladder cancer	Yes	15	3.7%	99	1.5%	3.18E-03	2.5 (1.4-4.5)
	No	389		6,548			

Only significant results were reported. *The number of missing data is 2,508 in location of cancer, 3,448 in TNM clinical classification: N, 3,787 in TNM clinical classification: M, 2,200 in estrogen-receptor status, 2,276 in progesterone-receptor status, and 3,952 in triple negative breast cancer, respectively.

Supplementary Table 5: Comparison of carrier frequency between cases and controls in men

Gene	No. of pathogenic variants	Case (n = 53)		Control (n = 12,490)				
		No. of carriers	Carrier frequency (%)	No. of carriers	Carrier frequency (%)	P value	OR	(95% CI)
BRCA2	26	10	18.87	26	0.21	1.73.E-16	111.2	(44.9-256.8)
CDH1	3	2	3.77	5	0.04	3.63.E-04	97.6	(9.1-612.9)
BRCA1	8	1	1.89	11	0.09	0.050	21.8	(0.5-154.6)
ATM	15	1	1.89	19	0.15	0.081	12.6	(0.3-82.4)
CHEK2	3	0	0	27	0.22	1	0	(0.0-35.5)
NBN	4	0	0	4	0.03	1	0	(0.0-362.9)
NF1	5	0	0	5	0.04	1	0	(0.0-262.1)
PALB2	3	0	0	3	0.02	1	0	(0.0-576.9)
PTEN	3	0	0	3	0.02	1	0	(0.0-576.9)
STK11	1	0	0	1	0.01	1	0	(0.0-8036.8)
TP53	4	0	0	25	0.20	1	0	(0.0-38.6)
Sum	75	13*	24.53	129	1.03	1.64E-14	31.1	(14.9-61.0)

*One patient had two pathogenic variants in *BRCA2* and *CDH1*.

Supplementary Table 6: Comparison of carrier frequencies of genes between this study and a large scale study using 35,409 women (Cancer 123: 1721-)

	This study		Cancer 123: 1721-				
Gene	N of carriers	Proportion	N of carriers	Proportion	P value	OR	(95% CI)
ATM	12	0.38%	329	0.93%	9.26.E-04 †	0.4	(0.2-0.7)
BRCA1	79	2.52%	814	2.30%	0.421	1.1	(0.9-1.4)
BRCA2	128	4.08%	828	2.34%	2.77.E-08 †	1.8	(1.5-2.2)
CDH1	2	0.06%	23	0.06%	1.000	1.0	(0.1-4.0)
CHEK2	12	0.38%	397	1.12%	2.34.E-05 †	0.3	(0.2-0.6)
NBN	0	0.00%	59	0.17%	0.014	0.0	(0.0-0.7)
PALB2	14	0.45%	316	0.89%	8.14.E-03	0.5	(0.3-0.8)
PTEN	9	0.29%	17	0.05%	1.34.E-04 †	6.0	(2.4-14.2)
STK11	0	0.00%	4	0.01%	1.000	0.0	(0.0-17.1)
TP53	9	0.29%	61	0.17%	0.182	1.7	(0.7-3.4)
Sum*	263	8.39%	2,848	8.04%	0.494	1.0	(0.9-1.2)

Proportion of carriers between genes were significanlty different (chi-square test, P = 1.92 x 10^{-16}). *NF1* was not reported in Cancer 123: 1721-. *Double carrier was considered only in this study. † Significant after the Bonferroni correction was applied.

Supplementary Table 7: Gene and transcripts analyzed in this study

Gene	CCDS ID	Length of transcriptome (bp)	Total length analyzed (bp)[*2]
ATM	CCDS31669[*1]	9,171	9,419
BRCA1	CCDS11453	5,592	5,750
	CCDS11454	2,280	
	CCDS11455	2,100	
	CCDS11456[*1]	5,655	
	CCDS11459	5,451	
BRCA2	CCDS9344[*1]	10,257	10,361
CDH1	CCDS10869[*1]	2,649	2,713
CHEK2	CCDS13843	1,632	1,821
	CCDS13844	1,545	
	CCDS33629[*1]	1,761	
	CCDS58798	969	
NBN	CCDS6249[*1]	2,265	2,329
NF1	CCDS11264	8,457	8,813
	CCDS42292[*1]	8,520	
	CCDS45645	1,782	
PALB2	CCDS32406[*1]	3,561	3,613
PTEN	CCDS31238[*1]	1,212	1,248
STK11	CCDS45896[*1]	1,302	1,338
TP53	CCDS11118[*1]	1,182	1,311
	CCDS45605	1,041	
	CCDS45606	1,026	
Sum			48,716

[*1]All transcripts were used to determine annotation of each variant and higher effect was selected if multiple trancsripts caused different effects. We reported the longest transcript in each gene as representative in the manuscript, figures, and tables, when multiple transcrpts caused resulted in the same effect at different protein positions.

[*2]Total length includes all transcripts and 2 bp flanking sequences.

Supplementary Table 8: Descriptive summary of the 1,781 variants

	Common (5%≦MAF)	Low frequency (1%≦MAF<5%)	Rare (MAF<1%)	Sum
Total number of variants	30	27	1,724	1,781
Number of disruptive variants (%)[*1]	0 (0%)	0 (0%)	210 (12.2%)	210 (11.8%)
Number of nonsynonymous variants (%)	13 (43.3%)	20 (74.1%)	1,051 (61.0%)	1,084 (60.9%)
Number of synonymous variants (%)	17 (56.7%)	7 (25.9%)	463 (26.9%)	487 (27.3%)
Number of novel variants (%)[*2]	0 (0%)	0 (0%)	949 (55.0%)	949 (53.3%)

[*1]Disruptive variants includes nonsense variants, splice site variants, and frameshift variants predicted by SnpEff.

[*2]Not registered in the dbSNP build 147.

Supplementary Table 9: Criteria for classifying pathogenic variants in this study

Impact	ACMG/AMP Criteria		Procedure used in this study
Very strong	PVS1	Null variant (nonsense, frameshift, canonical ±1 or 2 splice sites, initiation codon, single or multiexon deletion) in a gene where LOF is a known mechanism of disease	Effect of each variant on amino acid sequence was assigned by the SnpEff (ver. 4.2) according to the 28 transcripts registered in CCDS (ver. 15). When one variant had several effects caused by different transcripts, higher effect was selected. If effect was HIGH including nonsense, frameshift, and splice site variants, PVS1 = 1.
Strong	PS1	Same amino acid change as a previously established pathogenic variant regardless of nucleotide change	If amino acid change is same as a pathogenic (without conflict) variant in the ClinVar on 4/Nov/2016 but nucleotide change is different from it, PS1=1.
	PS2	De novo (both maternity and paternity confirmed) in a patient with the disease and no family history	This category was not used because we did not use trio samples.
	PS3	Well-established in vitro or in vivo functional studies supportive of a damaging effect on the gene or gene product	We selected variants with a functional test showing a deleterious effect on the gene and gene product registed in the HGMD database (ver. 2016.1). If two researchers agreed with its interpretation, PS3 = 1.
	PS4	The prevalence of the variant in affected individuals is significantly increased compared with the prevalence in controls	According to Note 1 of Table 3 in the ACMG/AMP guidelines (Genet Med 17; 405-), if association analysis showed OR > 5 and its confidential interval >1, PS4 = 1.
Moderate	PM1	Located in a mutational hot spot and/or critical and well-established functional domain (e.g., active site of an enzyme) without benign variation	The coordinates of Pfam domains in each gene were downloaded from the UCSC genome browser table function. If a variant was located in this domain, PM1 = 1.
	PM2	Absent from controls (or at extremely low frequency if recessive) in Exome Sequencing Project, 1000 Genomes Project, or Exome Aggregation Consortium	If this variant is not found in either ExAC without TCGA samples nor ToMMo (https://ijgvd.megabank.tohoku.ac.jp/author/tommo/), PM2=1.
	PM3	For recessive disorders, detected in trans with a pathogenic variant	This category was not used because we did not use trio samples.
	PM4	Protein length changes as a result of in-frame deletions/insertions in a nonrepeat region or stop-loss variants	If a variant was in-frame deletion/insertion located outside of Simple Repeat in the UCSC browser, PM4 = 1.
	PM5	Novel missense change at an amino acid residue where a different missense change determined to be pathogenic has been seen before	If a variant caused different amino acid change at the same location as pathogenic variants used in PS1, PM5 = 1
	PM6	Assumed de novo, but without confirmation of paternity and maternity	This category was not used because we did not use trio samples.
Supporting	PP1	Cosegregation with disease in multiple affected family members in a gene definitively known to cause the disease	This category was not used because we did not use multiple affected family members.
	PP2	Missense variant in a gene that has a low rate of benign missense variation and in which missense variants are a common mechanism of disease	This catergory was not used. Although association between protein-truncating variants of the 11 genes and breast cancer risk has been established, there was not enough evidence for pathogenicity about missense variants.
	PP3	Multiple lines of computational evidence support a deleterious effect on the gene or gene product (conservation, evolutionary, splicing impact, etc.)	We used FATHMM and KGGSeq because they showed the best deleteriousness prediction for nonsynonymous variants (Hum Mol Genet 24;2125-, 2015). We obtained predictions of FATHMM (ver. 3.0a) and KGGSeq(ver. 1.0+) by ANNOVA. If predications were D for FATHMM and Yes for KGGSeq, PP3 = 1.
	PP4	Patient's phenotype or family history is highly specific for a disease with a single genetic etiology	This category was not used according to AJHG 98; 801-.
	PP5	Reputable source recently reports variant as pathogenic, but the evidence is not available to the laboratory to perform an independent evaluation	This category was not used because we only used ClinVar.

Supplementary Table 10: Criteria for classifying benign variants in this study

Impact		ACMG/AMP Criteria	Criteria used in this study
Stand-alone	BA1	Allele frequency is >5% in Exome Sequencing Project, 1000 Genomes Project, or Exome Aggregation Consortium	We used frequencies of five populations in the 1000 genomes project phase 3, six populations in the ExAC project, and the ToMMo. If frequency of a variant is > 1% in ≥ 1 population based on the ENIGMA BRCA1/2 Gene Variant Classification Criteria (v2.4), BA1 = 1.
Strong	BS1	Allele frequency is greater than expected for disorder	If frequency of a variant is between 0.1% and 1% in ≧1 population in the same dataset as BA1 based on the ENIGMA BRCA1/2 Gene Variant Classification Criteria (v2.4), BS1 = 1.
	BS2	Observed in a healthy adult individual for a recessive (homozygous), dominant (heterozygous), or X-linked (hemizygous) disorder, with full penetrance expected at an early age	This category was not used because variants in the 11 genes were not known full penetrant at 60 years old.
	BS3	Well-established in vitro or in vivo functional studies show no damaging effect on protein function or splicing	We selected variants with a functional test not showing a deleterious effect on the gene and gene product registered in the HGMD database. If two researchers agreed with its interpretation, BS3 = 1.
	BS4	Lack of segregation in affected members of a family	This category was not used because we did not use multiple affected family members.
Supporting	BP1	Missense variant in a gene for which primarily truncating variants are known to cause disease	This catergory was not used. Although association between protein-truncating variants of the 11 genes and breast cancer risk has been established, there was not enough evidence for benign about missense variants.
	BP2	Observed in trans with a pathogenic variant for a fully penetrant dominant gene/disorder or observed in cis with a pathogenic variant in any inheritance pattern	This category was not used because we did not use trio samples.
	BP3	In-frame deletions/insertions in a repetitive region without a known function	If a variant was in-frame deletion/insertion located inside of Simple Repeat in the UCSC browser, BP3 = 1.
	BP4	Multiple lines of computational evidence suggest no impact on gene or gene product (conservation, evolutionary, splicing impact, etc.)	If predications were T for FATHMM and No for KGGSeq, BP4 = 1.
	BP5	Variant found in a case with an alternate molecular basis for disease	If a variant was observed in cases who had a pathogenic variant already registered in ClinVar, BP5 = 1.
	BP6	Reputable source recently reports variant as benign, but the evidence is not available to the laboratory to perform an independent evaluation	This category was not used because we only used ClinVar.
	BP7	A synonymous (silent) variant for which splicing prediction algorithms predict no impact to the splice consensus sequence nor the creation of a new splice site AND the nucleotide is not highly conserved	If variant satisfied the following three criteria, BP7 = 1. Putative impact by SnpEff was LOW; dpsi_max_tissue of Spidex (ver. 1.0) provided by ANNOVAR was between -5% and 5% (Science 347; 144-); Mamml Cons in UCSC browser was <1.6 (Hum Mol Genet 24; 2125-).

Supplementary Table 11: Comparison of clinical significance determined by the ACMG/AMP guidelines in this study with the ClinVar in women

Clinical significance in ClinVar		Clinival significance determined by the ACMG/AMP guidelines in this study						
	3 tier	Pathogenic (224 variants)		Benign (227 variants)		Uncertain significance (1,330 variants)		
	5 tier	Pathogenic	Likely pathogenic	Benign	Likely benign	Uncertain significance (both evidence)	Uncertain significance (insufficient evidence)	Sum
3 tier	Clinical significance							
Pathogenic (113 variants)	Pathogenic	6	36	0	0	0	10	52
	Pathogenic,not provided	4	32	0	0	0	2	38
	Pathogenic,other	1	4	0	0	0	3	8
	Pathogenic/Likely pathogenic	1	4	0	0	0	2	7
	Pathogenic/Likely pathogenic,not provided	0	1	0	0	0	0	1
	Pathogenic/Likely pathogenic,not provided,other	0	1	0	0	0	0	1
	Pathogenic/Likely pathogenic,other	0	1	0	0	0	0	1
	Likely pathogenic	0	2	0	0	0	3	5
Benign (246 variants)	Benign	0	0	33	1	0	1	35
	Benign,not provided	0	0	1	0	0	2	3
	Benign,not provided,other	0	0	1	1	0	0	2
	Benign,other	0	0	8	4	0	0	12
	Benign/Likely benign	0	0	5	20	0	10	35
	Benign/Likely benign,not provided	0	0	0	0	0	1	1
	Benign/Likely benign,other	0	0	3	4	0	2	9
	Benign/Likely benign,other,not provided	0	0	1	1	0	0	2
	Likely benign	0	0	1	30	0	106	137
	Likely benign,not provided	0	0	0	1	0	0	1
	Likely benign,not provided,other	0	0	0	1	0	2	3
	Likely benign,other	0	0	0	1	0	5	6
Uncertain significance (409 variants)	Uncertain significance	0	6	0	40	0	222	268
	Uncertain significance,not provided	1	1	0	4	0	35	41
	Uncertain significance,other	0	0	1	3	0	2	6
	Conflicting interpretations of pathogencity	0	4	2	17	2	28	53
	Conflicting interpretations of pathogencity,drug response	0	0	1	0	0	0	1
	Conflicting interpretations of pathogencity,not provided	0	1	1	2	0	3	7
	Conflicting interpretations of pathogencity,not provided,other	0	0	0	1	0	2	3
	Conflicting interpretations of pathogencity,other	1	1	3	4	0	14	23
	not provided	0	3	0	0	0	3	6
	other	0	0	0	0	0	1	1
Not registered (1,013 variants)	Not registered	3	110	1	30	0	869	1,013
Sum	Sum	17	207	62	165	2	1,328	1,781

Supplementary Table 12: Variants detected in women whose clinical significance was uncertain significance detemined by the ACMG/AMP guidelines but pathogenic in the ClinVar

chr	Pos	Ref	Alt	SNP name	Gene	Effect	HGVS.c	HGVS.p	Carrier frequency in 7,051 cases	Carrier frequency in 11,241 controls	P value	OR	(95% CI)	Review status in ClinVar
10	89,685,308	A	G	.	*PTEN*	missense_variant	c.203A>G	p.Tyr68Cys	0.00014	0	0.385	Inf	(0.0-Inf)	Single submitter
11	108,216,545	C	T	rs587779872	*ATM*	missense_variant	c.8494C>T	p.Arg2832Cys	0	0.00009	1.000	0.0	(0.0-62.1)	Multiple submitters
13	32,903,604	CTG	C	rs768580992	*BRCA2*	frameshift_variant	c.658_659delGT	p.Val220fs	0.00014	0	0.385	Inf	(0.0-Inf)	Reviewed by expert panel
13	32,907,014	A	T	rs80358427	*BRCA2*	stop_gained	c.1399A>T	p.Lys467*	0.00014	0	0.385	Inf	(0.0-Inf)	Reviewed by expert panel
13	32,914,137	C	A	rs80358785	*BRCA2*	stop_gained	c.5645C>A	p.Ser1882*	0.00071	0.00018	0.116	4.0	(0.7-41.9)	Reviewed by expert panel
13	32,930,687	C	T	rs80358981	*BRCA2*	stop_gained	c.7558C>T	p.Arg2520*	0.00043	0	0.057	Inf	(0.7-Inf)	Reviewed by expert panel
13	32,937,362	A	G	rs397507954	*BRCA2*	missense_variant	c.8023A>G	p.Ile2675Val	0.00043	0.00009	0.163	4.8	(0.4-250.8)	Multiple submitters
13	32,937,504	C	A	.	*BRCA2*	missense_variant	c.8165C>A	p.Thr2722Lys	0.00014	0	0.385	Inf	(0.0-Inf)	Single submitter
13	32,968,951	C	T	rs80359212	*BRCA2*	stop_gained	c.9382C>T	p.Arg3128*	0.00043	0.00009	0.163	4.8	(0.4-250.8)	Reviewed by expert panel
16	23,614,911	GAAGT	G	rs587776424	*PALB2*	frameshift_variant	c.3426_3429delACTT	p.Leu1142fs	0.00014	0	0.385	Inf	(0.0-Inf)	Multiple submitters
16	23,641,218	G	A	rs180177110	*PALB2*	stop_gained	c.2257C>T	p.Arg753*	0.00014	0	0.385	Inf	(0.0-Inf)	Multiple submitters
16	23,647,108	T	TA	rs756660214	*PALB2*	frameshift_variant	c.758dupT	p.Ser254fs	0.00014	0	0.385	Inf	(0.0-Inf)	Multiple submitters
17	7,577,139	G	A	rs55832599	*TP53*	missense_variant	c.799C>T	p.Arg267Trp	0.00028	0	0.149	Inf	(0.3-Inf)	Single submitter
17	29,670,115	CTAACTT	C	rs864622639	*NF1*	inframe_deletion	c.7159_7164delAAC TTT	p.Asn2387_Phe2388del	0.00014	0	0.385	Inf	(0.0-Inf)	Single submitter
17	41,242,961	C	T	rs80356857	*BRCA1*	splice_region_varian t&synonymous_varia	c.4185G>A	p.Gln1395Gln	0.00014	0	0.385	Inf	(0.0-Inf)	Multiple submitters
17	41,256,190	G	T	rs80356888	*BRCA1*	stop_gained	c.390C>A	p.Tyr130*	0.00028	0	0.149	Inf	(0.3-Inf)	Reviewed by expert panel
17	41,267,797	C	T	rs80358018	*BRCA1*	splice_acceptor_vari ant&intron_variant	c.81-1G>A		0.00028	0	0.149	Inf	(0.3-Inf)	Multiple submitters
22	29,121,230	C	T	rs121908698	*CHEK2*	splice_donor_variant &intron_variant	c.573+1G>A		0.00014	0	0.385	Inf	(0.0-Inf)	Multiple submitters
22	29,121,266	G	A	rs730881701	*CHEK2*	stop_gained	c.538C>T	p.Arg180*	0.00014	0.00018	1.000	0.8	(0.0-15.3)	Multiple submitters
22	29,130,427	G	A	rs587781269	*CHEK2*	stop_gained	c.283C>T	p.Arg95*	0	0.00009	1.000	0.0	(0.0-62.1)	Multiple submitters

Supplementary Table 13: Comparison of clinical significance determined by the ACMG/AMP guidelines with the clinical significance recorded in ClinVar in men

Clinical significance in ClinVar		Clinival significance determined by the ACMG/AMP guidelines in this study						
	3 tier	Pathogenic (66 variants)		Benign (176 variants)		Uncertain significance (1,028 variants)		
	5 tier	Pathogenic	Likely pathogenic	Benign	Likely benign	Uncertain significance (both evidence)	Uncertain significance (insufficient evidence)	Sum
3 tier	Clinical significance							
Pathogenic (34 variants)	Pathogenic	2	11	0	0	0	6	19
	Pathogenic,not provided	5	4	0	0	0	2	11
	Pathogenic,other	0	1	0	0	0	0	1
	Pathogenic/Likely pathogenic	1	0	0	0	0	0	1
	Pathogenic/Likely pathogenic,other	0	1	0	0	0	0	1
	Likely pathogenic	0	0	0	0	0	1	1
Benign (221 variants)	Benign	0	0	32	1	0	2	35
	Benign,not provided	0	0	1	0	0	2	3
	Benign,not provided,other	0	0	1	0	0	1	2
	Benign,other	0	0	10	3	0	1	14
	Benign/Likely benign	0	0	5	12	0	12	29
	Benign/Likely benign,not provided	0	0	0	0	0	1	1
	Benign/Likely benign,not provided,other	0	0	0	1	0	0	1
	Benign/Likely benign,other	0	0	4	7	0	1	12
	Benign/Likely benign,other,not provided	0	0	1	1	0	0	2
	Likely benign	0	0	1	18	0	95	114
	Likely benign,not provided	0	0	0	1	0	0	1
	Likely benign,not provided,other	0	0	0	2	0	1	3
	Likely benign,other	0	0	0	0	0	4	4
Uncertain significance (325 variants)	Uncertain significance	0	2	0	29	0	194	225
	Uncertain significance,not provided	0	0	0	1	0	27	28
	Uncertain significance,other	0	0	1	3	0	2	6
	Conflicting interpretations of pathogencity	0	3	2	17	0	19	41
	Conflicting interpretations of pathogencity,drug response	0	0	1	0	0	0	1
	Conflicting interpretations of pathogencity,not provided	0	0	1	1	0	4	6
	Conflicting interpretations of pathogencity,not provided,other	0	0	0	1	0	1	2
	Conflicting interpretations of pathogencity,other	0	1	3	3	0	7	14
	not provided	0	0	0	0	0	2	2
Not registered (690 variants)	Not registered	1	34	0	12	1	642	690
Sum	Sum	9	57	63	113	1	1,027	1,270

Supplementary Table 14: Comparison of proportion of cases with pathogenic variants categorised as high-risk versus low-risk patients according to NCCN guidelines

		Patients with pathogenic variants			
Criteria	Number of patients	Number	Proportion	95% CI	P value*
[Patients with low-risk for further genetic risk evaluation]	1,164	41	3.5%	2.5-4.7%	Reference
[Patients with High-risk for further genetic risk evaluation - all]	3,136	268	8.5%	7.6-9.6%	1.98.E-08
[Patients with High-risk for further genetic risk evaluation - split by criterion met]					
Age at breast cancer diagnosis ≤ 50 years old	2,413	199	8.2%	7.2-9.4%	1.94E-07
Triple negative breast cancer diagnosed at ≤ 60 years old	193	26	13.5%	9.0-19.1%	1.44E-06
Bilateral breast cancer	124	20	16.1%	10.1-23.8%	1.41E-06
Combined with pancreatic cancer at any age	3	1	33.3%	0.8-90.6%	0.132
≥ 1 family member with breast cancer at any age	834	94	11.3%	9.2-13.6%	2.85E-10
≥ 1 family member with ovarian cancer at any age	83	19	22.9%	14.4-33.4%	2.56E-08
≥ 2 family members with breast and/or pancreatic cancer at any age	104	23	22.1%	14.6-31.3%	2.66E-09
[Patients with undetermined-risk]	2,751	95	3.5%	2.8-4.2%	0.924

*P value from Fisher's exact test comparing proportion of pathogenic variants with the low-risk category patients.

Supplementary Table 15: Comparison of clinical significance in 1,781 variants

		Use of female and male controls		
		Pathogenic	Benign	Uncertain significance
Use of only female controls	Pathogenic	244	0	0
	Benign	0	356	0
	Uncertain significance	1	7	1,173

Supplementary Table 16: Result of gene-based association test using pathogenic variants in 7,051 cases and 23,731 female and male controls.

Gene	No. of pathogenic variants	Case (n = 7,051) No. of carriers (%)	Control (n = 23,731) No. of carriers (%)	P value	OR	(95% CI)
BRCA2	92	191 (2.71)	44 (0.19)	1.23×10^{-80}	15.0	(10.7-21.3)
BRCA1	57	102 (1.45)	17 (0.07)	6.40×10^{-48}	20.5	(12.2-36.5)
PALB2	23	28 (0.40)	9 (0.04)	1.53×10^{-11}	10.5	(4.8-25.3)
TP53	15	16 (0.23)	6 (0.03)	9.92×10^{-7}	9.0	(3.3-28.1)
CHEK2	18	26 (0.37)	23 (0.10)	7.25×10^{-6}	3.8	(2.1-7.0)
PTEN	15	11 (0.16)	4 (0.02)	4.84×10^{-5}	9.3	(2.7-39.9)
ATM	39	26 (0.37)	39 (0.16)	0.003	2.2	(1.3-3.8)
NF1	13	8 (0.11)	5 (0.02)	0.003	5.4	(1.6-20.9)
CDH1	3	2 (0.03)	1 (0.00)	0.133	6.7	(0.4-396.2)
NBN	7	1 (0.01)	7 (0.03)	0.692	0.5	(0.0-3.7)
STK11	2	0 (0.00)	2 (0.01)	1	0.0	(0.0-17.9)
Sum	284	408 (5.79)	157 (0.66)	5.99×10^{-133}	8.7	(7.2-10.6)

Supplementary Figure 1: The number of known and novel pathogenic variants

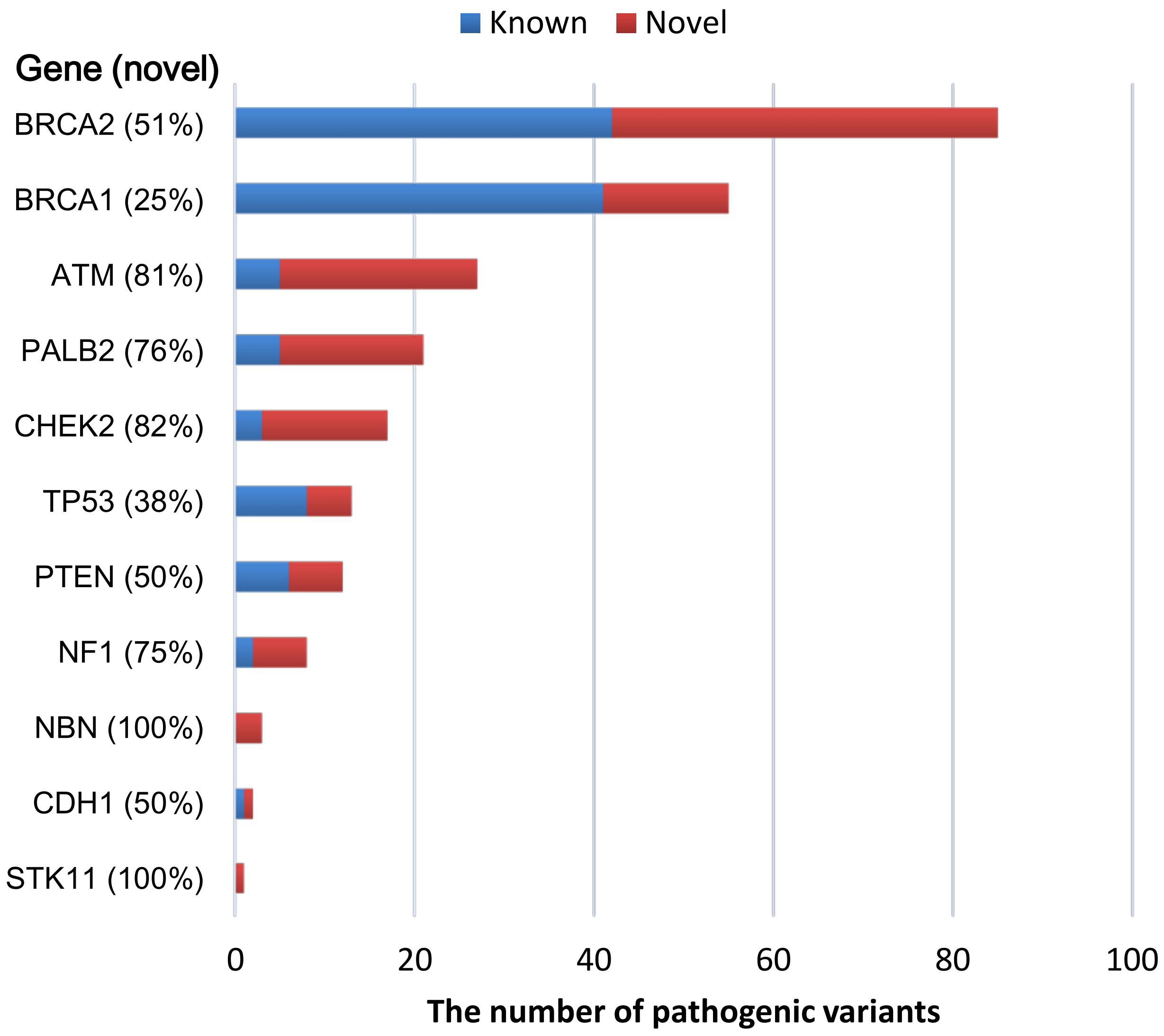

Variants not registered in the ClinVar on 4/Nov/2016 are considered novel.

Supplementary Figure 2: Location and the number of pathogenic variants in Japanese breast cancer women

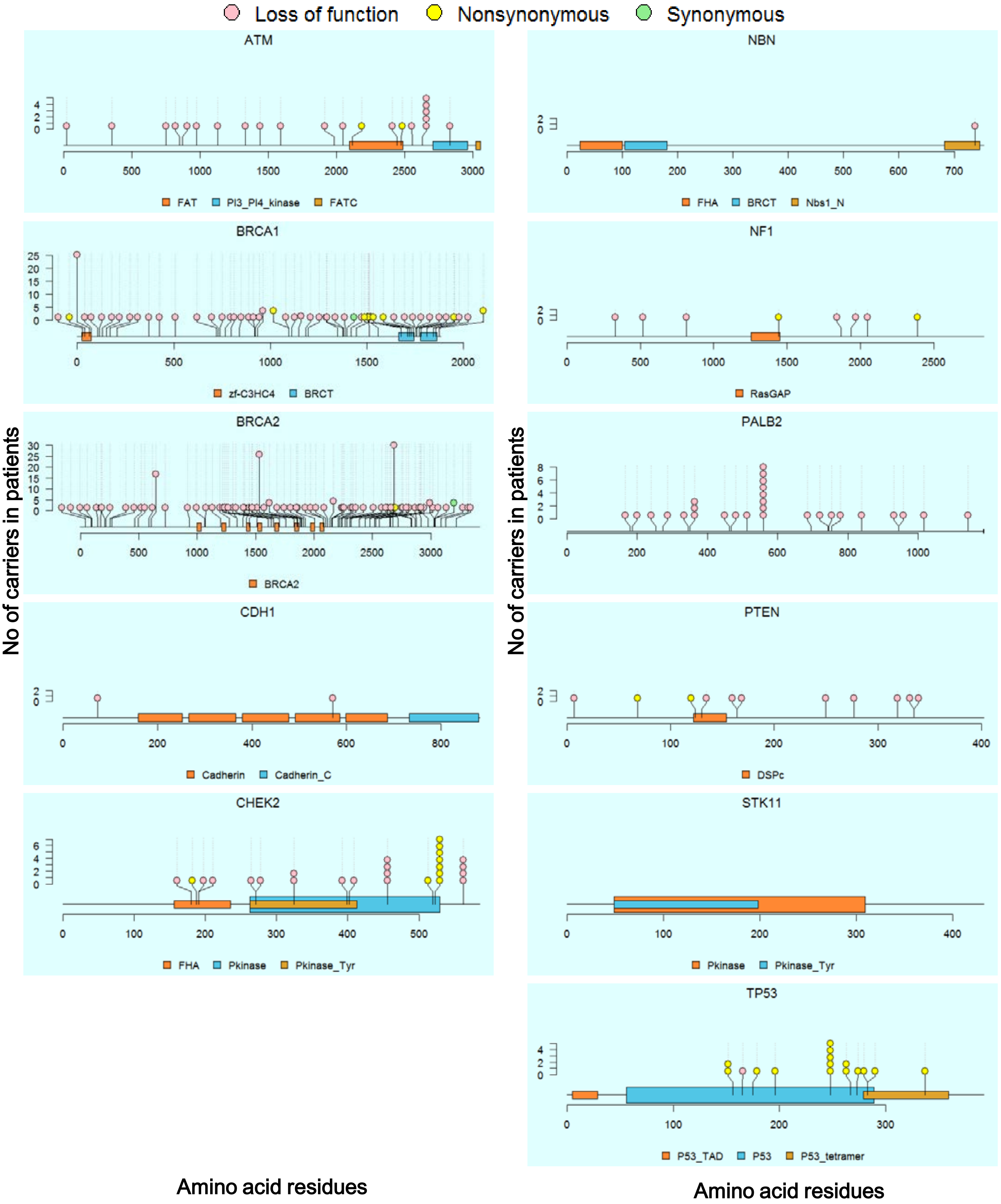

Locations of pathogenic variants found in patients and domains in proteins encoded by the six genes are shown by lollipop structures, with the variant type indicated by color. Pink, yellow, and green circle indicates loss of function, nonsynonymous, and synonymous variants, respectively. The x axis reflects the number of amino acid residues, and the y axis shows the total number of patients with each pathogenic variant.

Supplementary Figure 3: Proportion of genes impacted by pathogenic variants in female patients

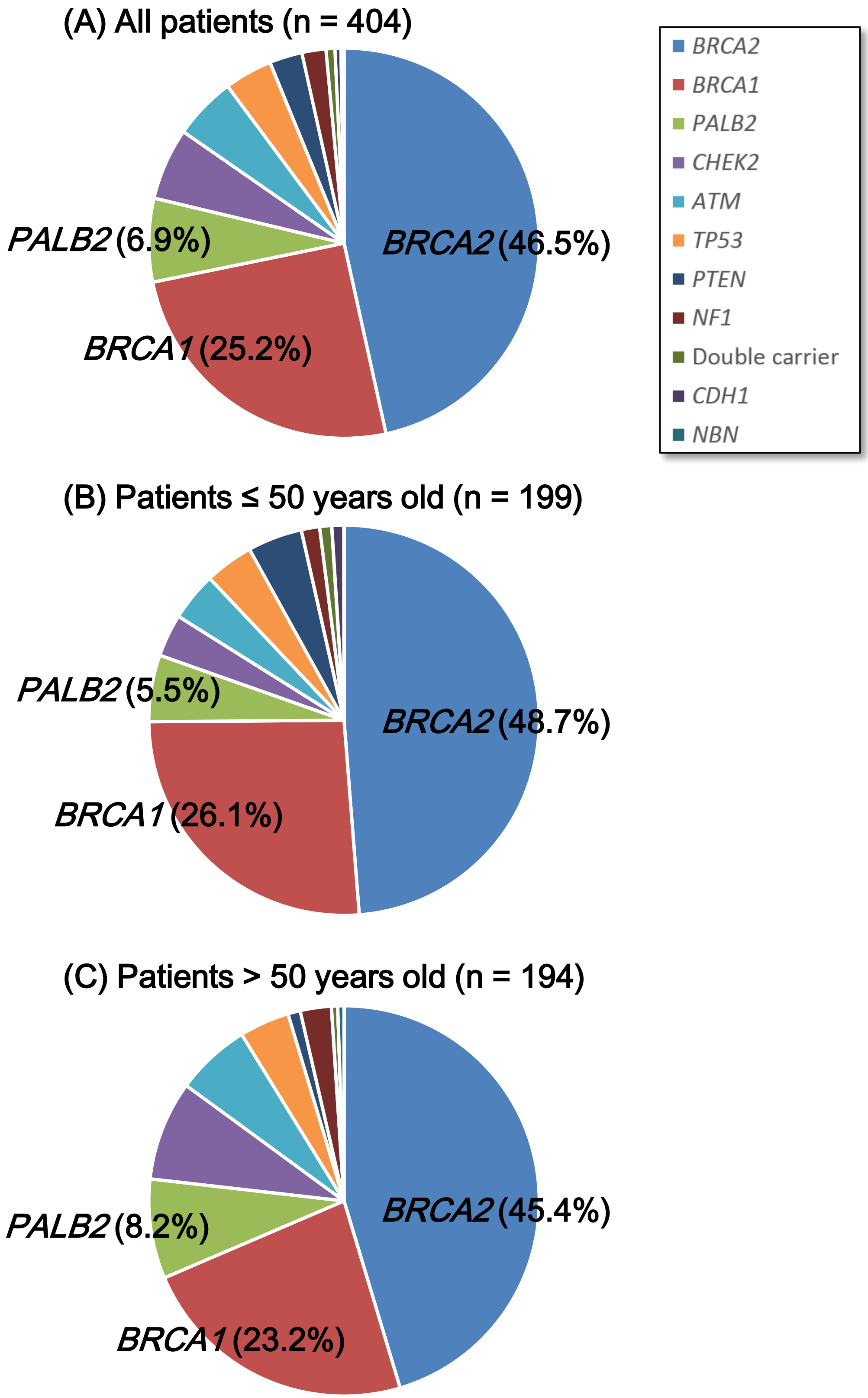

These pie charts show the proportion of genes detected in (A) 404 patients diagnosed at all ages, (B) 199 patients diagnosed at 50 years old and younger, and (C) 194 patients diagnosed at older than 50 years old. There was not significant differences (chi-square test, P = 0.155). Eleven patients were not included in pie charts of (B) or (C) due to the missing data of age at diagnosis.

Supplementary Figure 4: Location and the number of pathogenic variants in *BRCA2* in unselected Japanese breast cancer men

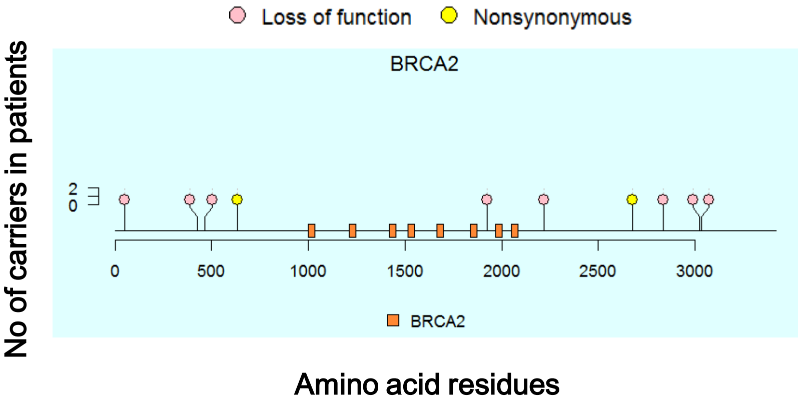

Locations of pathogenic variants found in patients and domains in proteins encoded by *BRCA2* are shown by lollipop structures, with the variant type indicated by color. Pink and yellow circle indicates loss of function, and nonsynonymous, respectively. The x axis reflects the number of amino acid residues. All pathogenic variants were found in one patient only.

Supplementary Figure 5: The number of patients with known and novel pathogenic variants

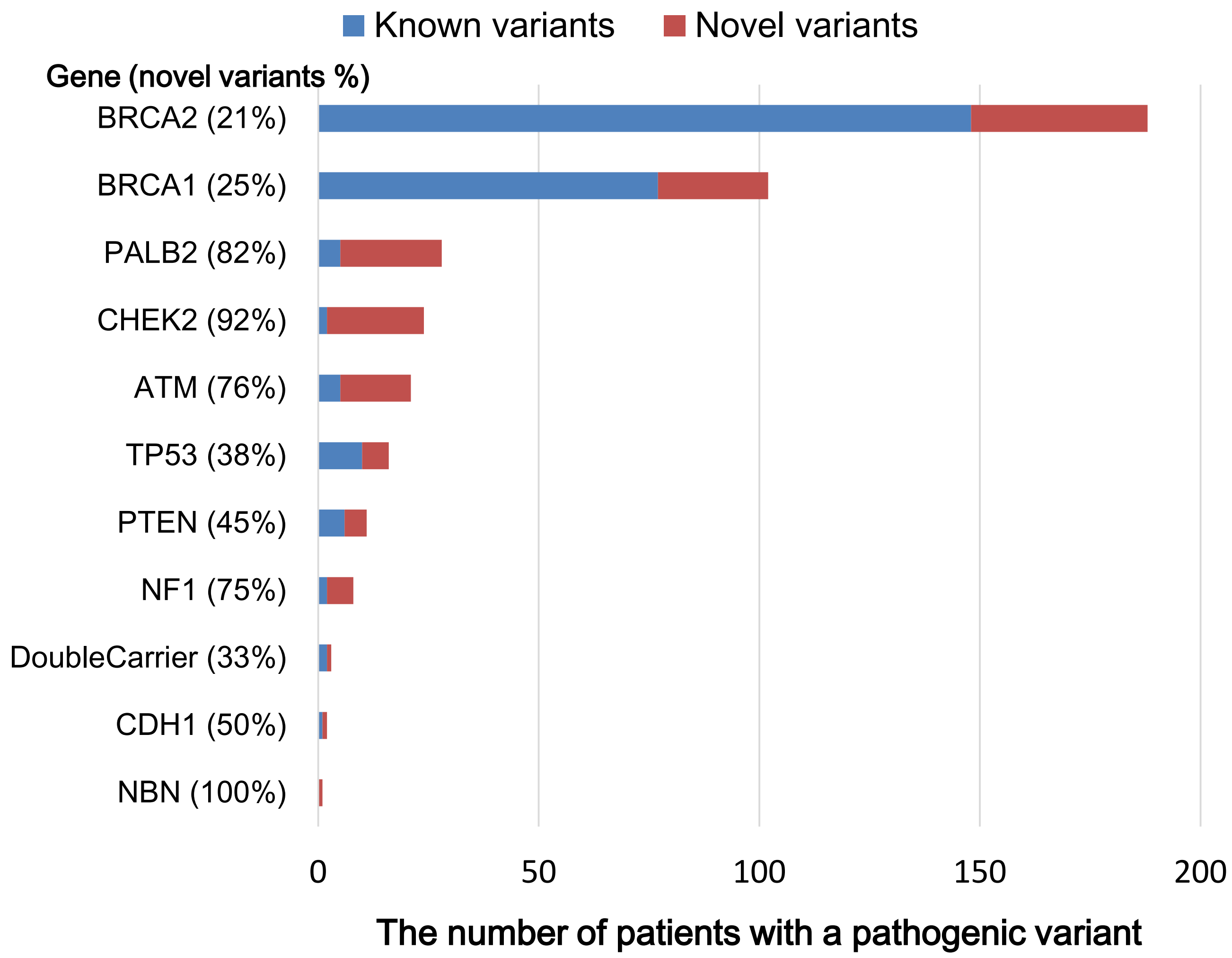

Variants not registered in the ClinVar on 4/Nov/2016 are considered novel.

Supplementary Figure 6: Correlation between length of the target region and number of variants for each gene in women

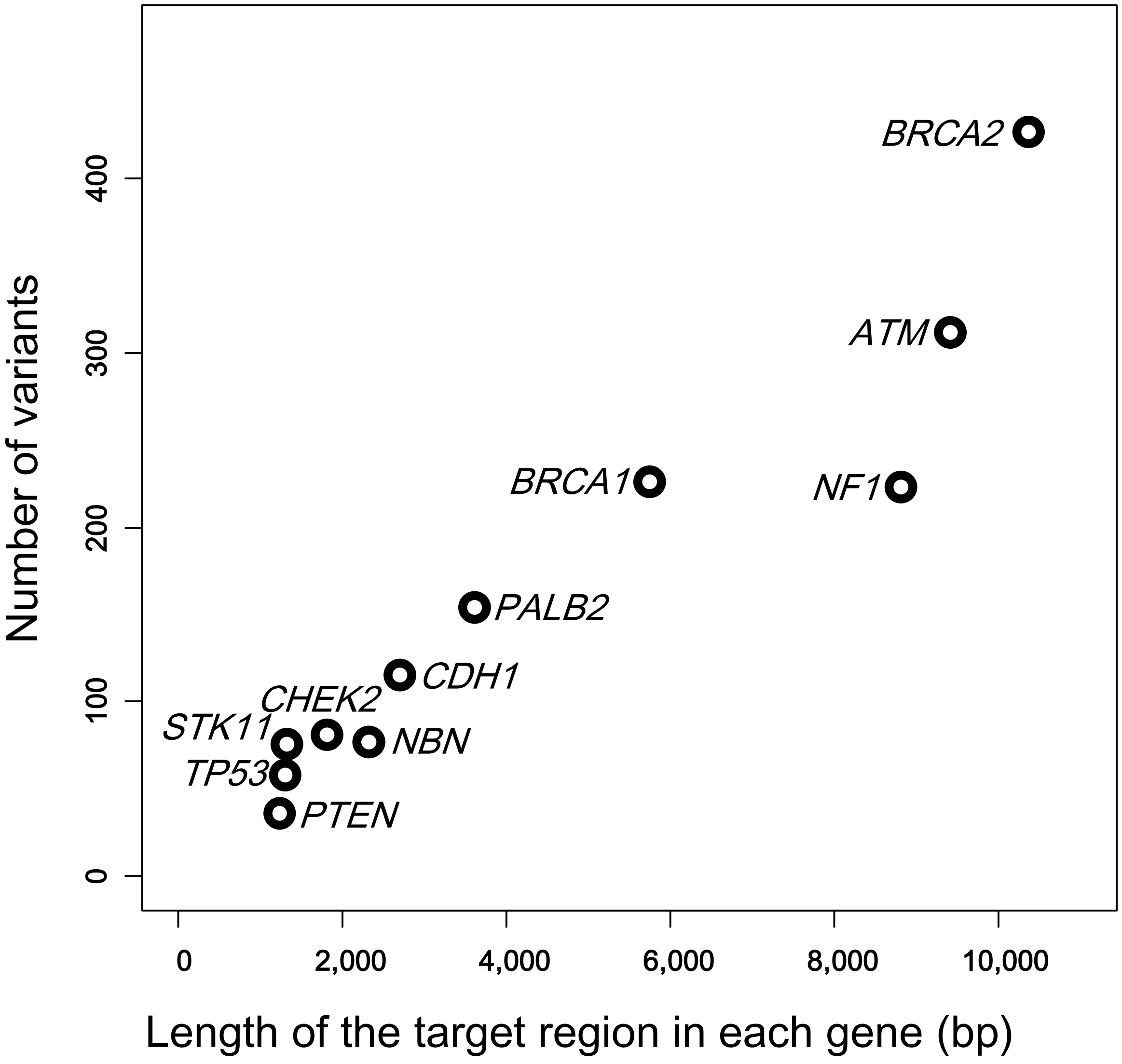

The number of variants was strongly correlated with the gene length (Pearson correlation coefficient, r = 0.953, p = 5.70 x 10^{-6}).

Supplementary References

1. Sherry ST*, et al.* dbSNP: the NCBI database of genetic variation. *Nucleic Acids Res.* **29**, 308-311 (2001).
2. Strande NT*, et al.* Evaluating the Clinical Validity of Gene-Disease Associations: An Evidence-Based Framework Developed by the Clinical Genome Resource. *Am. J. Hum. Genet.* **100**, 895-906 (2017).
3. Richards S*, et al.* Standards and guidelines for the interpretation of sequence variants: a joint consensus recommendation of the American College of Medical Genetics and Genomics and the Association for Molecular Pathology. *Genet. Med.* **17**, 405-424 (2015).
4. Maxwell KN*, et al.* Evaluation of ACMG-Guideline-Based Variant Classification of Cancer Susceptibility and Non-Cancer-Associated Genes in Families Affected by Breast Cancer. *Am. J. Hum. Genet.* **98**, 801-817 (2016).
5. Spurdle AB*, et al.* ENIGMA--evidence-based network for the interpretation of germline mutant alleles: an international initiative to evaluate risk and clinical significance associated with sequence variation in BRCA1 and BRCA2 genes. *Hum. Mutat.* **33**, 2-7 (2012).

6. Landrum MJ*, et al.* ClinVar: public archive of interpretations of clinically relevant variants. *Nucleic Acids Res.* **44**, D862-868 (2016).

7. 1000 Genomes Project Consortium*, et al.* An integrated map of genetic variation from 1,092 human genomes. *Nature* **491**, 56-65 (2012).

8. Lek M*, et al.* Analysis of protein-coding genetic variation in 60,706 humans. *Nature* **536**, 285-291 (2016).

9. Nagasaki M*, et al.* Rare variant discovery by deep whole-genome sequencing of 1,070 Japanese individuals. *Nat. Commun.* **6**, 8018 (2015).

10. Shihab HA, Gough J, Cooper DN, Day IN, Gaunt TR. Predicting the functional consequences of cancer-associated amino acid substitutions. *Bioinformatics* **29**, 1504-1510 (2013).

11. Li MX, Gui HS, Kwan JS, Bao SY, Sham PC. A comprehensive framework for prioritizing variants in exome sequencing studies of Mendelian diseases. *Nucleic Acids Res.* **40**, e53 (2012).

12. Xiong HY*, et al.* RNA splicing. The human splicing code reveals new insights into the genetic determinants of disease. *Science* **347**, 1254806 (2015).

13. Stenson PD, Mort M, Ball EV, Shaw K, Phillips A, Cooper DN. The Human

Gene Mutation Database: building a comprehensive mutation repository for clinical and molecular genetics, diagnostic testing and personalized genomic medicine. *Hum. Genet.* **133**, 1-9 (2014).

14. Cingolani P*, et al.* A program for annotating and predicting the effects of single nucleotide polymorphisms, SnpEff: SNPs in the genome of Drosophila melanogaster strain w1118; iso-2; iso-3. *Fly (Austin)* **6**, 80-92 (2012).

15. Farrell CM*, et al.* Current status and new features of the Consensus Coding Sequence database. *Nucleic Acids Res.* **42**, D865-872 (2014).

16. Eilbeck K, Quinlan A, Yandell M. Settling the score: variant prioritization and Mendelian disease. *Nat. Rev. Genet.* **18**, 599-612 (2017).

17. Daly MB*, et al.* Genetic/familial high-risk assessment: breast and ovarian, version 1.2014. *J. Natl. Compr. Canc. Netw.* **12**, 1326-1338 (2014).

18. Buys SS*, et al.* A study of over 35,000 women with breast cancer tested with a 25-gene panel of hereditary cancer genes. *Cancer* **123**, 1721-1730 (2017).

19. Pritzlaff M*, et al.* Male breast cancer in a multi-gene panel testing cohort: insights and unexpected results. *Breast Cancer Res. Treat.* **161**, 575-586

(2017).

20. Slavin TP*, et al.* The contribution of pathogenic variants in breast cancer susceptibility genes to familial breast cancer risk. *NPJ Breast Cancer* **3**, 22 (2017).
