# Supplementary material for: Germline pathogenic variants of 11 breast cancer genes in 7,051 Japanese patients and 11,241 controls
Source: Nat Commun. 2018 Oct 4;9:4083. doi: 10.1038/s41467-018-06581-8 (PMC6172276; doi:10.1038/s41467-018-06581-8)
Supplement: Supplementary file 3 — Description of Additional Supplementary Files [file 41467_2018_6581_MOESM3_ESM.docx]

**Description of Additional Supplementary Files**

File Name: Supplementary Data 1

Description: A list of all 1,781 variants and its clinical significance in women.

File Name: Supplementary Data 2

Description: A list of all 1,270 variants and its clinical significance in men.
